# Loss of Shp1 impairs myeloid cell function and causes lethal inflammation in zebrafish larvae

**DOI:** 10.1242/dmm.049715

**Published:** 2023-01-30

**Authors:** Maaike Allers, Petra A. Bakker, Jelmer Hoeksma, Herman P. Spaink, Jeroen den Hertog

**Affiliations:** ^1^ Hubrecht Institute-KNAW and University Medical Center Utrecht, 3584 CT Utrecht, The Netherlands; ^2^ Institute Biology Leiden, Leiden University, 2333 BE Leiden, The Netherlands

**Keywords:** Shp1, Macrophage, Neutrophil, Protein-tyrosine phosphatase, Zebrafish

## Abstract

*PTPN6* encodes SHP1, a protein tyrosine phosphatase with an essential role in immune cell function. SHP1 mutations are associated with neutrophilic dermatoses and emphysema in humans, which resembles the phenotype seen in *motheaten* mice that lack functional SHP1. To investigate the function of Shp1 in developing zebrafish embryos, we generated a *ptpn6* knockout zebrafish line lacking functional Shp1. Shp1 knockout caused severe inflammation and lethality around 17 days post fertilization (dpf). During early development, the myeloid lineage was affected, resulting in a decrease in the number of neutrophils and a concomitant increase in the number of macrophages. The number of emerging hematopoietic stem and progenitor cells (HSPCs) was decreased, but due to hyperproliferation, the number of HSPCs was higher in *ptpn6* mutants than in siblings at 5 dpf. Finally, the directional migration of neutrophils and macrophages was decreased in response to wounding, and fewer macrophages were recruited to the wound site. Yet, regeneration of the caudal fin fold was normal. We conclude that loss of Shp1 impaired neutrophil and macrophage function, and caused severe inflammation and lethality at the larval stage.

## Teaser

**Summary:** Loss of Shp1 in zebrafish impairs neutrophil and macrophage function and results in severe inflammation and lethality during the larval stage.

## INTRODUCTION

The non-receptor protein tyrosine phosphatase SHP1, encoded by the *PTPN6* gene, is a key regulator of immune cell function. SHP1 consists of two N-terminal Src homology 2 (SH2) domains, a catalytic domain and a C-terminal regulatory tail, and is mainly expressed in hematopoietic cells (Neel et al., 2003). Because of its important role in regulating immune cell function, SHP1 has become an interesting target for the treatment of auto-immune diseases and cancer in recent years (Watson et al., 2016).

SHP1 function has been studied extensively in the context of the *motheaten* (*me/me*) mouse, which has a spontaneous recessive mutation in *Ptpn6*. This mutation creates a cryptic splice site, which results in the loss of functional SHP1 (Tsui et al., 1993). The homozygous mutation leads to immune deficiency, widespread inflammation, skin lesions and death within 2-6 weeks due to lethal pneumonitis, characterized by the infiltration of myeloid cells (Green and Shultz, 1975). In the decades following the initial identification of the *me/me* mouse, multiple alternative less lethal mutations in *Ptpn6* have been described. *motheaten viable* has a mutation in a splice consensus site of *Ptpn6*, which results in alternative splicing. SHP1 from *motheaten viable* mice exhibits 80% reduction in phosphatase activity, and *motheaten viable* mice die by 9-12 weeks (Shultz et al., 1984). *spin* mice have a Y208N mutation in the C-terminal SH2 domain of SHP1, resulting in 50% residual catalytic activitym, and they die after more than a year (Croker et al., 2008). The symptoms of *motheaten viable* mice are highly similar to the symptoms of *motheaten* mice, although they develop lethal pneumonitis approximately 8 weeks later. *spin* mutants do not show lethal pneumonitis or immunodeficiency. This is likely due to the higher residual phosphatase activity of mutant SHP1 in *spin* mice. Heterozygous missense and splice variant mutations in *PTPN6* have also been found in human patients. These mutations are associated with emphysema and neutrophilic dermatoses (Nesterovitch et al., 2011; Bossé et al., 2019).

Compound mouse knockout lines and conditional knockout strains have been generated to study the function of depletion of SHP1 in specific cell types. Mouse double knockouts lacking RAG1 and SHP1 still show the *motheaten* phenotype, which indicates that the acquired immune system is not essential for the symptoms caused by the loss of SHP1 (Yu et al., 1996). Conditional neutrophil-specific knockout of SHP1 does not recapitulate the lethal pneumonitis phenotype nor the autoimmunity that is observed in the complete SHP1 knockout. Yet, knockout of SHP1 in the neutrophil-specific lineage causes dermal inflammation. Knockout of SHP1 in the dendritic cell lineage does not cause inflammation, but it does cause lymphadenopathy and autoimmunity (Abram et al., 2013; Abram and Lowell, 2017), indicating that depletion of SHP1 in different cell types results in distinct hematologic defects.

Zebrafish do not have a functional acquired immune system until the larvae are 4-6 weeks old (Lam et al., 2004). During early development, zebrafish only have innate immunity. In addition, zebrafish provide the opportunity to investigate embryonic development from the start, due to their translucent eggs and embryos. Therefore, zebrafish is the ideal model system to investigate the function of Shp1 in the innate immune system in a whole organism.

Previous morpholino-mediated knockdown studies in zebrafish embryos showed that Shp1 knockdown reduced the ability of embryos to combat infection with *Salmonella typhimurium* and *Mycobacterium marinum* (Kanwal et al., 2013). The innate immune system was hyperactivated to a counterproductive level. Experiments suggest that Shp1 functions as a negative regulator that imposes a tight control over the level of innate immune response activation.

Here, we developed a genetic zebrafish model lacking functional Shp1 to study the hematopoietic system during early development in the absence of Shp1. Zebrafish mutant for Shp1 recapitulated the lethality and inflammation seen in *motheaten* mice. In addition, we investigated the development of hematopoietic stem and progenitor cells (HSPCs) and all major blood lineages. We found that, during early development, macrophage numbers were increased, whereas neutrophil numbers were decreased. The emergence of HSPCs was reduced, but their subsequent proliferation was strongly increased. Finally, we show that the recruitment of macrophages and neutrophils to wound sites was disturbed in Shp1 mutants. Our results indicate that Shp1 has a role in hematopoietic development from the start until the development of specific cell types of the myeloid lineage, and Shp1 has an essential role in myeloid behavior after development as well.

## RESULTS

### Shp1 knockout leads to inflammation and is lethal at late larval stages

**Fig. 1.**
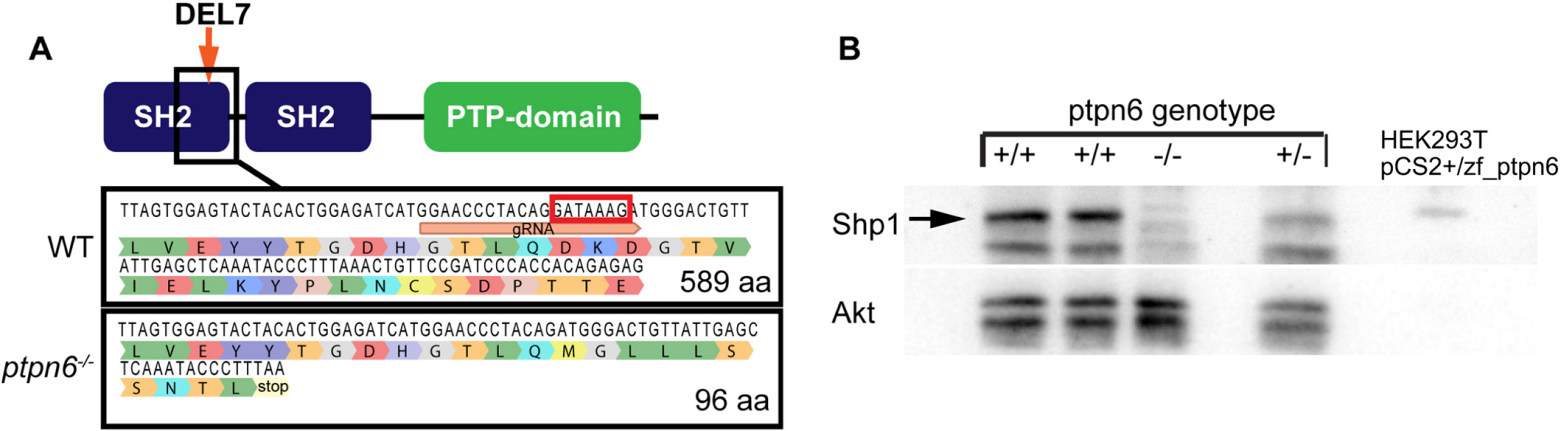
**The zebrafish *ptpn6* mutant generated using CRISPR/Cas9 is a strong null allele.** (A) Schematic overview of Shp1. gRNA was targeted to the C-terminal end of the N-terminal SH2 domain, inducing a 7 bp deletion (DEL7) in exon 4, indicated by the red box in the WT sequence. The mutation was confirmed by Sanger sequencing after sub-cloning of F1 DNA. The 7 bp deletion leads to a frameshift and a stop codon in exon 4, ten amino acids downstream of the mutation site. (B) Immunoblots detecting endogenous Shp1 in WT and heterozygous embryos, but not in homozygous *ptpn6* mutant embryos. Lysates of transfected HEK293T cells expressing zebrafish Shp1 were used as a control for the detection of zebrafish Shp1. An Akt-specific antibody was used to monitor equal loading.

We generated mutations in the zebrafish *ptpn6* gene using CRISPR-Cas9 technology at the one-cell stage, followed by screening of the F0 generation for germline mutations. We identified a 7 bp deletion in exon 4, resulting in a frame shift and a premature stop codon ten amino acids downstream of the mutation site. Exon 4 encodes the C-terminal part of the N-terminal SH2 domain, positioning the mutation upstream of the catalytic PTP domain. This deletion predicts the production of a severely truncated protein containing 96 amino acids (Fig. 1A). Reverse transcription PCR and sequencing indicated that mRNAs with the 7 bp deletion were produced in the mutant zebrafish line. To confirm the absence of the Shp1 protein, we analyzed total Shp1 protein levels in 5 days post fertilization (dpf) zebrafish embryo lysates with a zebrafish Shp1-specific antibody that we generated (Fig. 1B). At 5 dpf, *ptpn6* mutants showed no obvious phenotype (Fig. S1), in contrast to previous morpholino studies, which showed pleiotropic defects from 2 dpf onwards (Kanwal et al., 2013).

**Fig. 2.**
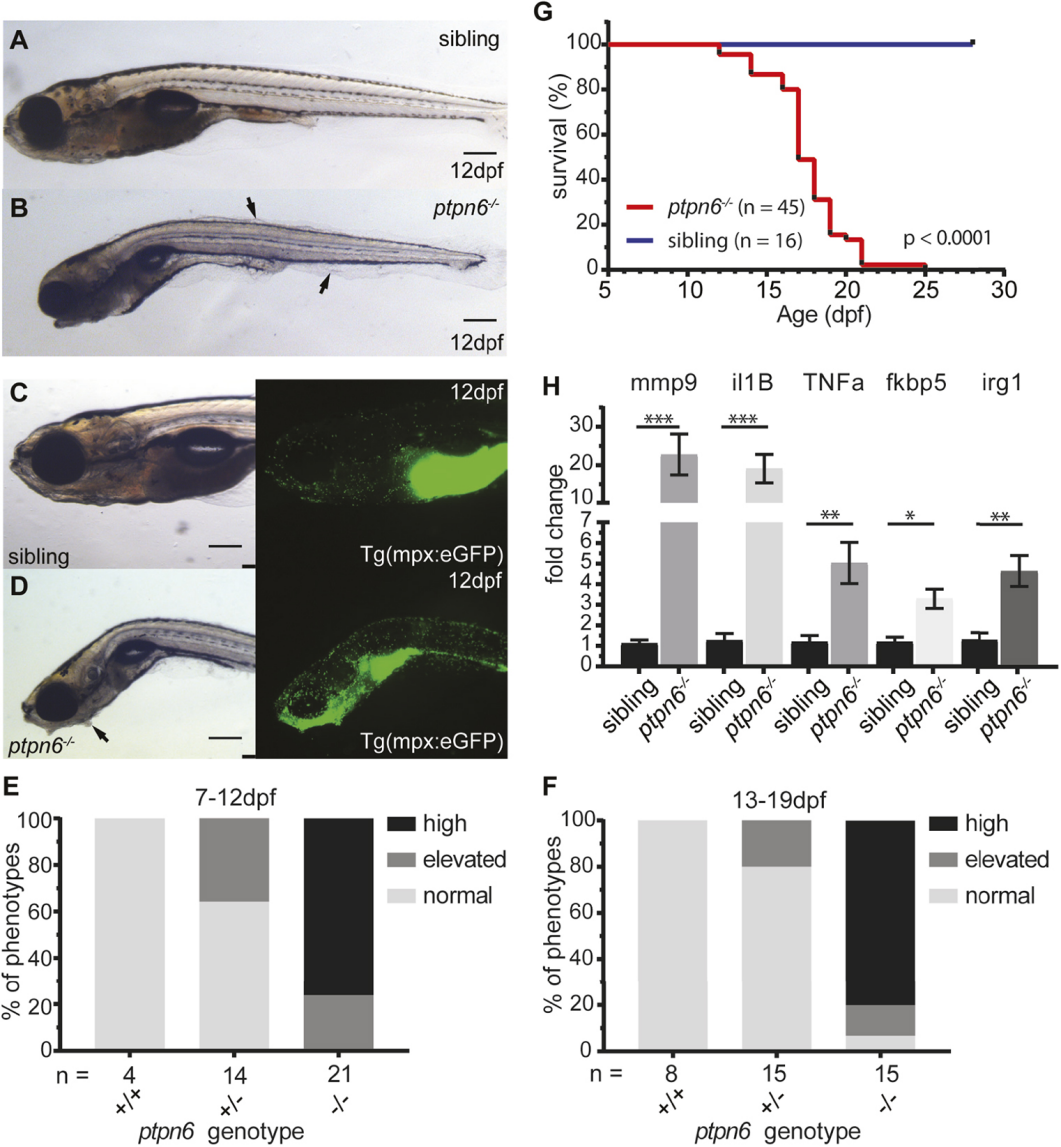
**Zebrafish *ptpn6* mutants show an inflammatory response with neutrophil accumulation and do not survive to adulthood.** (A-D) Representative pictures of embryos at 12 dpf by stereo microscopy. The larvae were genotyped by PCR and sequencing following imaging. Siblings (A,C) and *ptpn6^−/−^* mutants (B,D) displaying the *motheaten*-like phenotype. Arrows indicate the affected epithelia. GFP-positive neutrophils in *tg(mpx:eGFP)* transgenic lines (C,D, right) localize to the gill and mandibular area in *ptpn6^−/−^* embryos. Scale bars: 0.5 mm (A-D). (E,F) Scoring of phenotypes in age cohorts of larvae. Larvae with an affected phenotype were selected along with age-matched controls and the number of neutrophils was scored by eye. Subsequently, the larvae were genotyped by PCR and sequencing. Larvae were classified as normal (distribution and number of neutrophils as in WT larvae), elevated (mildly increased number of neutrophils in gill and mandibular area) or high (massive increase of neutrophil numbers in the gill and mandibular area). (G) Survival curve of *ptpn6* mutants. Larvae were monitored during raising, sacrificed at a defined end point (curved, skinny body, not able to swim upright and/or severe skin alterations) and genotyped by PCR and sequencing. Curve comparison was performed by log-rank (Mantel–Cox) test. (H) Increased expression of pro-inflammatory genes in mutants. RNA was extracted from larvae sacrificed upon observation of a severe phenotype (*n*=12) and their age-matched siblings (*n*=9). Gene expression was determined by qPCR and fold changes were calculated per time point (10, 14 and 18 dpf) and pooled afterwards. Statistical comparisons were performed by non-parametric ANOVA (Kruskal–Wallis) followed by multiple comparisons (original false discovery rate method of Benjamini–Hochberg). *P<0.05; **P<0.01; ***P<0.001. Error bars show the s.e.m.

During later larval stages, the *ptpn6* mutants appeared smaller and skinnier than their siblings and developed a curved phenotype. Moreover, as described for the *motheaten* mouse, the mutants showed abnormalities in their skin epithelia (ruffled epithelial edges and bumps) (Fig. 2A-D) (Green and Shultz, 1975). To look at infiltration of the skin with immune cells, we used the *Tg(mpx:eGFP)* line marking myeloid-specific peroxidase-producing neutrophils. We followed the developing larvae, mutants and siblings over time and imaged them either at the moment of sacrifice or at the end point of the experiment (either 12 or 19 dpf). Surprisingly, not only did we observe neutrophil infiltration of the skin, but the most prominent accumulation of neutrophils was found in the gill area (Fig. 2C,D). *motheaten* mice succumb to pneumonitis that is characterized by neutrophil and macrophage accumulation in the lungs (Green and Shultz, 1975). We quantified the neutrophil accumulation phenotype by scoring the number of neutrophils in the gill and mandibular area into three categories, namely, normal or wild type (WT), elevated and high, at 7-12 dpf or 13-19 dpf (Fig. 2E,F). It was evident that neutrophil numbers in the scored area were greatly increased in mutants.

In accordance with studies on the *Ptpn6^me/me^* and *Ptpn6^me-v/me-v^* mice (Green and Shultz, 1975; Shultz et al., 1984), *ptpn6* mutant zebrafish did not survive to adulthood. The absence of Shp1 was lethal at late larval stages, with a median survival of 17 days (Fig. 2G). To investigate whether affected larvae presented with inflammation, we collected mRNA from mutants (*n*=12) and age-matched siblings (*n*=9) at 10, 14 and 18 dpf, corresponding to the end points of the mutant larvae. We performed quantitative real-time PCR (qRT-PCR) for several pro-inflammatory genes and genes that are upregulated during inflammation (*mmp9*, *il1b*, *tnfa*, *fkbp5* and *irg1/acod1*), of which *mmp9* and *il1b* were previously found to be upregulated in the *ptpn6* morpholino studies in zebrafish embryos (Kanwal et al., 2013). All five genes were significantly upregulated compared to siblings (Fig. 2H), indicating that loss of functional Shp1 evokes an inflammatory response, although we cannot exclude the possibility that this response is caused by non-specific inflammation or necrosis.

### The development of erythroid and thrombocyte lineages is unaffected in *ptpn6* mutants

To assess erythropoiesis, we performed o-Dianisidine staining and *gata1a in situ* hybridization on 5 dpf embryos. o-Dianisidine detects heme in hemoglobin and *gata1* marks early erythrocytes (Iuchi and Yamamoto, 1983; Ransom et al., 1996). We found no difference in erythrocyte numbers or distribution between *ptpn6* mutants and siblings (Fig. S2A-E). To investigate the thrombocyte lineage, the nucleated equivalents of mammalian megakaryocytes/platelets, we quantified the number of thrombocytes (GFP^high^ cells) in *Tg(cd41:GFP)* embryos at 5 dpf by confocal microscopy. In these transgenic embryos, thrombocytes express a high level of GFP and HSPCs express a low level of GFP (Kissa et al., 2008). After fixation, only GFP^high^ cells were visible (Fig. S2F,G). Counting of GFP^high^ cells revealed no difference in thrombocyte numbers between siblings and *ptpn6* mutants (Fig. S2H). Hence, the erythroid and thrombocyte lineages in zebrafish embryos lacking functional Shp1 appear to be unaffected at 5 dpf.

### Development of the myeloid cell lineage, but not early lymphocyte development, is affected in *ptpn6* mutants

**Fig. 3.**
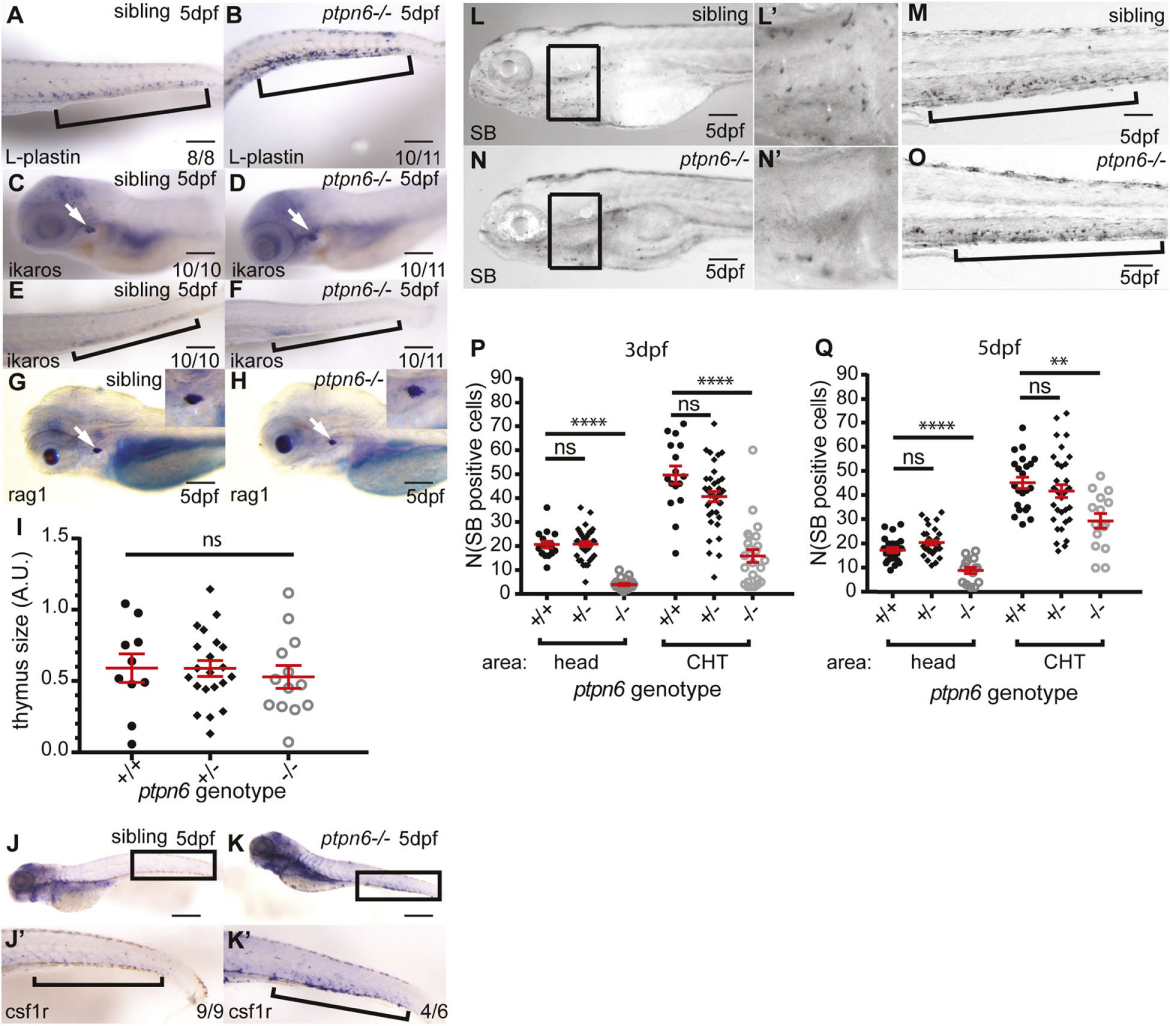
**Development of the leukocyte lineages in *ptpn6* mutants.** (A-H) A panel of *in situ* hybridization markers for leukocyte lineages was used on 5 dpf WT and *ptpn6^−/−^* embryos. The CHT is indicated by brackets and the thymus by white arrows. The number of embryos showing the depicted pattern/total number of embryos is shown in the bottom right corner. The localization of L-plastin (*lcp1*), pan-leukocyte marker (A,B); *ikaros*, lymphoid progenitor marker (C-F); and *rag1*, lymphocyte marker (G,H) was analyzed. Representative lateral views of the thymus are shown, with close ups of the thymus in the inset (G,H). (I) Quantification of *rag1*-positive area. One-way ANOVA no post hoc test because no significance was detected. A.U., arbitrary units. (J,K) Localization of *csf1r*, a macrophage marker. J′,K′ show close ups of the CHT. (L-O) Sudan Black (SB) staining of neutrophils in two parts of the embryo at 5 dpf. L′,N′ show magnifications of the boxed areas in L,N. (P,Q) SB-positive cells were counted in the head (mainly primitive wave) and CHT (mainly definitive wave) at 3 and 5 dpf. All embryos were genotyped after *in situ* hybridization or SB staining and imaging by PCR and sequencing. Statistical comparisons were performed by one-way ANOVA followed by Tukey's multiple comparisons for both areas per time point separately. ns, not significant; **P<0.01; ****P<0.0001. Error bars show the s.e.m. Scale bars: 0.2 mm (A-H); 0.5 mm (J,K); 0.2 mm (L-O).

To investigate the development of leukocytes in *ptpn6* mutants, we first determined L-plastin (encoded by *lcp1*) expression, a pan-leukocyte marker, by whole-mount *in situ* hybridization. *lcp1* expression was higher in the caudal hematopoietic tissue (CHT) (the equivalent of the mammalian embryonic liver, where HSPCs migrate to mature before they migrate to the location where they produce blood of all lineages for the rest of their life, i.e. head kidney for fish and bone marrow for mammals) of *ptpn6* mutants, indicating an increase in total leukocyte numbers (Fig. 3A,B). To determine which cell type was responsible for this increase, we performed *in situ* hybridization and staining, and used transgenic lines to quantify cell numbers. First, we investigated the expression of *ikaros* (also known as *ikzf1*) and *rag1* by *in situ* hybridization to determine whether the development of the lymphoid lineage was affected. *ikaros* is an early lymphocyte marker and *rag1* is essential for V(D)J recombination in maturing B and T cells (Willett et al., 1997; 2001). As expected, *ikaros* expression was detectable in the CHT and the thymus of 5 dpf embryos and no differences in expression level or location were observed between *ptpn6* mutants and siblings (Fig. 3C-F). Quantification of the *rag1*-positive area in mutants, heterozygous embryos and WT siblings revealed no difference in thymus size, a direct readout for developing B- and T-cell numbers in the thymus (Fig. 3G-I). We conclude that early development of the lymphocyte system is not affected in *ptpn6* mutants.

Next, we investigated the two main cell types in the myeloid lineage, the monocytes/macrophages and the neutrophils. Expression of *csf1ra*, a marker for macrophages, was upregulated in the CHT of *ptpn6* mutants at 5 dpf (Fig. 3J,K). Sudan Black (SB) stains the granules of immature and mature neutrophils and was used to quantify these in 3 and 5 dpf embryos. SB-positive cells in the head are mainly derived from the primitive wave at early time points in development, whereas SB-positive cells in the CHT are derived from the definitive wave (Le Guyader et al., 2008). SB-positive cells were counted in these two areas of the embryos, indicated by black boxes (the head, including the heart) and by brackets (the CHT) (Fig. 3L-O). Surprisingly, given the late larval phenotype with increased neutrophil numbers (Fig. 2C,D), at 3 dpf, neutrophil numbers in the head area of *ptpn6* mutants were reduced by approximately 80% compared to WT siblings, and by 65% in the CHT (Fig. 3P). At 5 dpf, these numbers were reduced to approximately 50% and 35%, respectively (Fig. 3Q). In addition to the SB staining, we quantified neutrophils in 3 dpf *Tg(mpx:GFP)* embryos, which facilitated counting of neutrophils in intact embryos. Mpx is a neutrophil marker that is expressed early in neutrophil differentiation, from the promyelocyte phase onwards (Bainton et al., 1971; Borregaard and Cowland, 1997; Kumar et al., 2010). The total number of *mpx*-positive neutrophils was reduced by 41% in *ptpn6* mutants compared to WT siblings. Because *mpx* is expressed from early neutrophil precursors onwards, this indicated that the whole neutrophil lineage was affected, and not just later steps in neutrophil maturation (Fig. S3A,B).

**Fig. 4.**
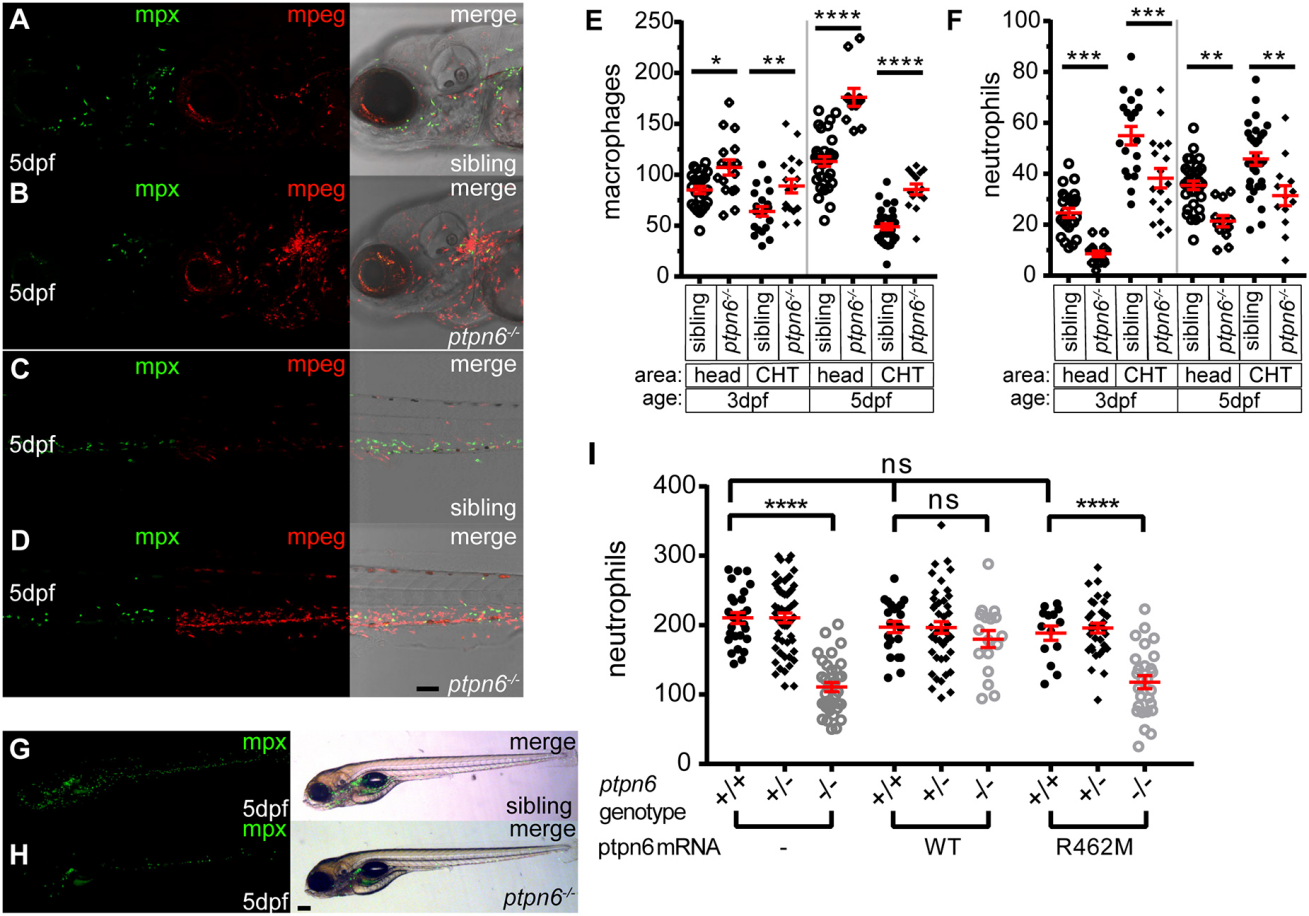
**Zebrafish *ptpn6* mutants have an increased number of macrophages and a reduced number of neutrophils, and this phenotype is dependent on the phosphatase activity of Shp1.** (A-D) Representative pictures of the head area (A,B) and of the anterior part of the CHT (C,D) of 5 dpf *tg(mpx:GFP/mpeg:mCherry) ptpn6* mutant and sibling embryos showing neutrophils and macrophages. Images were acquired using a 20× objective, a pinhole of 2 Airy units (AU) and a step size of 1.83 µm. (E,F) Quantification of macrophage (E) and neutrophil (F) numbers per embryo in the head and CHT of 3 and 5 dpf embryos, performed in ImageJ by particle analysis. Statistical comparisons by one-way ANOVA and Sidak's multiple comparison test for preselected columns. (G,H) Stereo images of 5 dpf *tg(mpx:GFP)* WT and *ptpn6^−/−^* non-injected embryos. Images of WT Shp1- and Shp1-R462M-injected embryos are shown in Fig. S4. (I) Quantification of the total number of neutrophils in *tg(mpx:eGFP)* embryos injected with mRNA encoding WT Shp1 or Shp1-R462M, showing the total neutrophil numbers at 5 days post injection per embryo. Quantification was performed in ImageJ by particle analysis. Following imaging, the embryos were genotyped by PCR and sequencing. Statistical comparisons were performed by one-way ANOVA and Tukey’s multiple comparisons test. Scale bars: 0.1 mm (A-D); 0.2 mm (G,H). *P<0.05, **P<0.01, ***P<0.001, ****P<0.0001. Error bars show the s.e.m.

To further investigate the development of the granulocyte-monocyte lineage, we used *Tg(mpx:GFP/mpeg1:mCherry)* transgenic fish to simultaneously quantify neutrophil and macrophage numbers. *mpeg1.1* expression is restricted to the monocyte/macrophage lineage during early development (Ellett et al., 2011). Using confocal imaging, we acquired images of the rostral part and the CHT of 3 and 5 dpf *ptpn6* mutant and sibling embryos (Fig. 4A-D; 3 dpf not shown). Macrophage (Fig. 4E) and neutrophil (Fig. 4F) numbers in both areas and at both time points were determined using ImageJ. Macrophage numbers were increased in both areas and at both time points during development, whereas neutrophil numbers were reduced. The increase in *mpeg* signal was very pronounced in the area of the pronephros, the site of late larval and adult hematopoiesis in zebrafish (Al-Adhami and Kunz, 1977; Willett et al., 1999), where macrophage numbers were up by ∼55%. Similarly, whereas peripheral neutrophils were scarcely found in the head, the pronephros was well populated with *mpx*-positive cells.

To exclude apoptosis as the cause of the reduction in neutrophils in *ptpn6* mutant embryos, we performed Acridine Orange staining. No increase in apoptosis was observed in the CHT of 5 dpf *ptpn6* mutant embryos (Fig. S4). These results suggest that *ptpn6* mutant fish suffer from an altered lineage balance within the myeloid cell lineage and early-onset increased proliferation in the macrophage lineage.

### The early developmental phenotype of the myeloid lineage is dependent on catalytic activity of Shp1

Next, we questioned whether restoring Shp1 expression rescued the phenotype observed in *ptpn6* mutant embryos. We micro-injected *Tg(mpx:GFP)* embryos at the one-cell stage with WT *ptpn6* mRNA and allowed them to develop until 5 dpf. The mRNA also encoded GFP, linked with a P2a autocatalytic cleavage sequence. Upon expression, GFP was visible until approximately 3 dpf, which allowed us to successfully select injected embryos at 1 dpf. Non-injected controls are depicted in Fig. 4G,H. Expression of WT Shp1 restored neutrophil numbers in *ptpn6* mutant embryos compared to those found in non-injected WT controls at 5 dpf (Fig. 4I; Fig. S5). Although catalytic activity is important for Shp1 function, other domains may exert alternative functions, e.g. scaffolding by SH2 domains (Timms et al., 1998; An et al., 2008; Abram and Lowell, 2017). To investigate whether the catalytic activity of Shp1 is essential for the development of neutrophils, we injected the catalytically inactive mutant Shp1-R462M. We used the R462M mutant and not the catalytic cysteine mutant C456S to avoid inadvertent substrate-trapping effects (Hale and den Hertog, 2018). Upon micro-injection of mRNA encoding GFP-P2a-Shp1-R462M, no increase in total neutrophil numbers was observed in *ptpn6* mutant embryos (Fig. 4I; Fig. S5). Expression of Shp1 or inactive Shp1-R462M did not affect the number of neutrophils in WT or heterozygous siblings (Fig. 4I). Therefore, we conclude that catalytic activity of the PTP domain of Shp1 is essential for its function in the development of the myeloid lineage.

### Early hematopoiesis is affected in *ptpn6* mutants

**Fig. 5.**
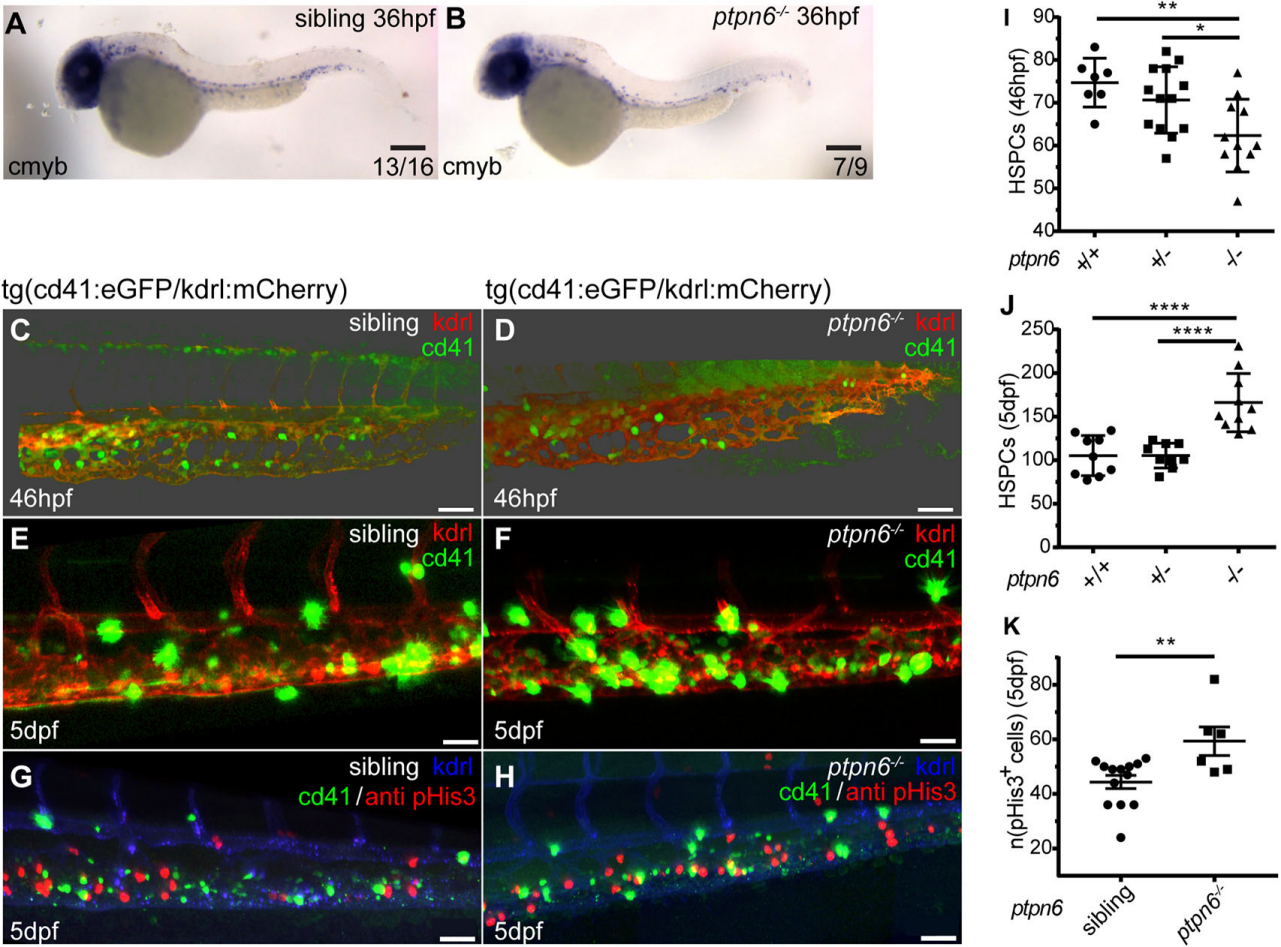
**HSPCs in *ptpn6* mutant embryos.** (A,B) Stereo images of *cmyb in situ* staining, an HSPC marker, at 36 hpf. The number of embryos showing the depicted pattern/total number of embryos is shown in the bottom right corner. Scale bars: 0.2 mm. (C-H) Confocal images of the CHT of *tg(cd41:eGFP/kdrl:mCherry)* zebrafish, acquired using a 40× objective and pinhole of 2 AU. The *cd41:eGFP* transgene marks thrombocytes at later stages (GFP^high^) and HSPCs (GFP^low^); the *kdrl:mCherry* transgene marks endothelial cells, including HSPCs that were derived from endothelial cells. (C,D) Embryos were selected and fixed at ∼46 hpf, when no GFP^high^ cells marking thrombocytes were present yet, only GFP^low^-positive HSPCs. Whole-mount immunohistochemistry was performed using a GFP-specific antibody. Representative 3D blended rendering images of the CHT of 46 hpf embryos are shown. Step size: 1.5 µm. Scale bars: 60 µm. (E,F) Live imaging of 5 dpf embryos. Representative 3D maximum-intensity projection images of part of the imaged region of the CHT are shown. Step size: 2 µm. Scale bars: 40 µm. (G,H) 5 dpf embryos were fixed and whole-mount immunohistochemistry was performed using a pHis3-specific (proliferation marker) and GFP-specific (GFP^low^ indicates HSPCs) antibody. Representative 3D maximum-intensity projection images of part of the imaged CHT are depicted. Step size: 2 µm. Scale bars: 40 µm. (I,J) Quantification of cells in confocal images using Imaris, indicating the numbers of GFP-positive HSPCs in the CHT of 46 hpf embryos (I) and GFP^low^ HSPCs in the CHT of 5 dpf embryos (J). Statistical comparisons were performed by one-way ANOVA followed by Tukey's multiple comparisons test. (K) Quantification of pHis3-positive cells in the CHT of 5 dpf embryos. All embryos were genotyped following *in situ* hybridization, immunohistochemistry and imaging by PCR and sequencing. Means were compared using a two-tailed unpaired Student's *t*-test. *P<0.05; **P<0.01; ****P<0.0001. Error bars show the s.e.m.

Earlier reports indicated that hematopoietic stem cells (HSCs) in *motheaten* mice are not affected by SHP1 deficiency (Shultz et al., 1983). However, recently, SHP1 was found to be involved in HSC quiescence in *Scl-CreER/Shp1^fl/fl^* mice (Jiang et al., 2018). Therefore, we investigated whether HSPC emergence, homing and proliferation was affected in *ptpn6* mutant zebrafish. First, the emergence and early homing of HSPCs at ∼36 hpf was investigated by *in situ* hybridization of *c-myb* (also known as *myb*) mRNA in *ptpn6* mutants and siblings (Fig. 5A,B). A minor decrease in signal was observed in *ptpn6* mutants. Subsequently *Tg(cd41:eGFP/kdrl:mCherry)* fish were used to quantify the number of GFP^low^ HSPCs in the CHT after arrival (Kissa et al., 2008; Choorapoikayil et al., 2014). *kdrl:mCherry*-positive embryos with red fluorescent endothelial cells were selected at 46 hpf, and subjected to immunohistochemistry with a GFP-specific antibody. Confocal imaging was used to capture the complete CHT, based on the *kdrl:mCherry* signal staining all endothelial cells of the CHT (Fig. 5C,D). Note that HSPCs that arrive at the CHT express *kdrl:mCherry* themselves as well, because these cells used to be endothelial cells in the floor of the dorsal aorta that underwent epithelial-to-hematopoietic transition (Kissa et al., 2008). The mCherry signal in HSPCs was hardly detectable in fixed embryos and the signal from the *cd41:eGFP* transgene was used to quantify the number of HSPCs. GFP-positive cells in the CHT were quantified and, at 46 hpf, a significant reduction in HSPCs in the CHT was observed in *ptpn6* mutant embryos compared to heterozygous embryos (*P*<0.05), and a 17% reduction compared to WT siblings (*P*<0.01) (Fig. 5I). Next, live imaging was performed of the CHT of 5 dpf *Tg(cd41:eGFP/kdrl:mCherry) ptpn6* mutant and sibling embryos (Fig. 5E,F). The number of GFP^low^ HSPCs was quantified, showing expansion of HSPCs in the CHT over time from 46 hpf to 5 dpf. The number of HSPCs was 58% higher in mutant embryos at 5 dpf than in heterozygous and WT embryos (*P*<0.0001) (Fig. 5J). To investigate the cause of the increase in HSPC number, proliferation of HSPCs was assessed in the CHT of *ptpn6* mutant embryos by immunostaining 5 dpf *Tg(cd41:eGFP/kdrl:mCherry)* embryos using pHis3- and GFP-specific antibodies (Fig. 5G,H). The Kdrl-mCherry signal was used to determine which cells were located in the CHT and the pHis3-positive cells were counted. A 34% increase in proliferating cells was detected in the CHT of *ptpn6* mutant embryos compared to sibling cell numbers (*P*<0.01) (Fig. 5K). Taken together, these results suggest that early HSPC emergence and/or arrival in the CHT is reduced or delayed in *ptpn6* mutant embryos, and that the proliferation of these definitive blood cell progenitors is enhanced once they seed the CHT.

### Directional migration of neutrophils and macrophages in response to tail wounding is affected in *ptpn6* mutants

**Fig. 6.**
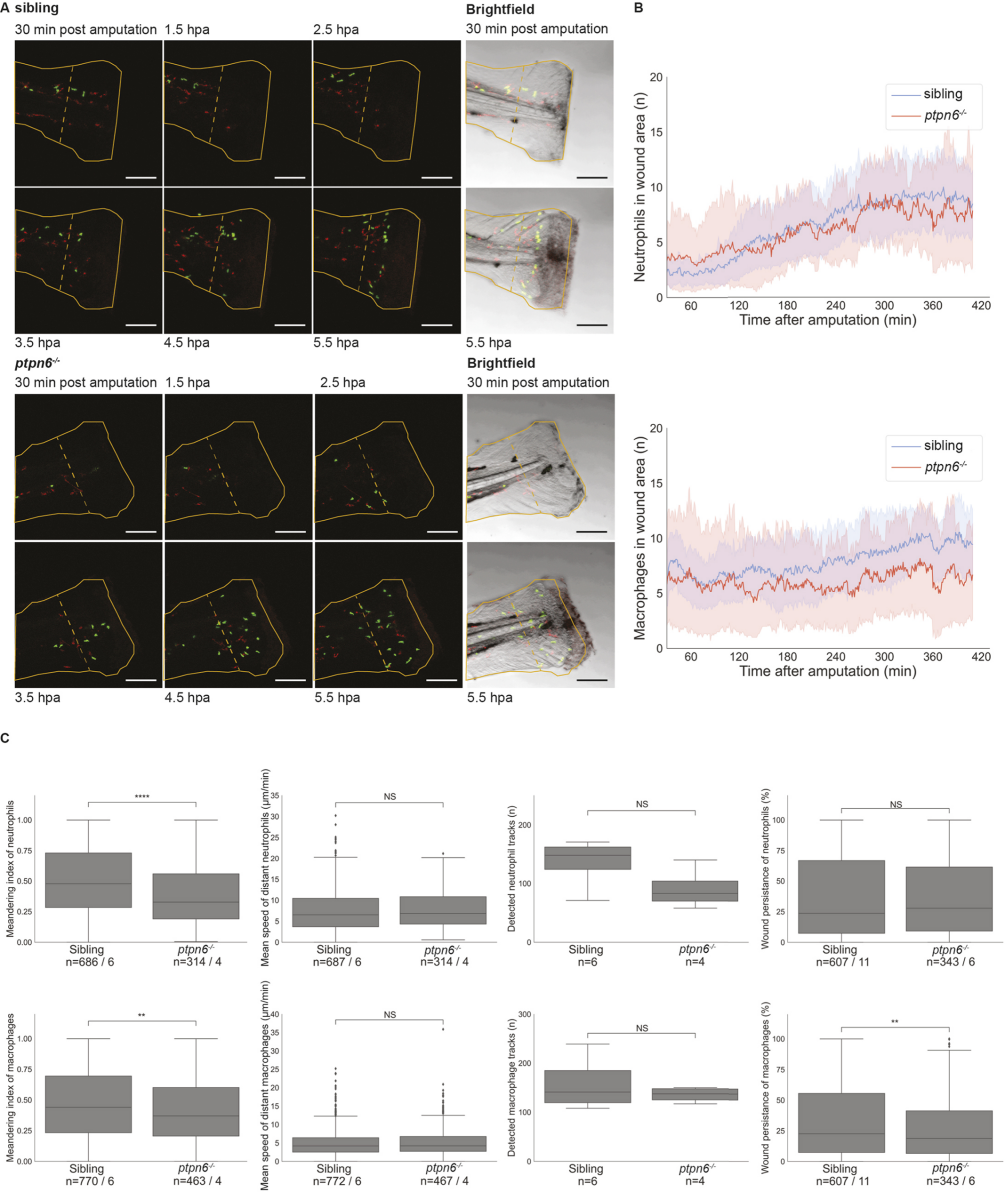
**Neutrophil and macrophage migration is affected upon tail fin fold amputation in Shp1 mutants.** Embryos were genotyped by PCR and sequencing following imaging. (A) Still images from live imaging of mutant and sibling 4 dpf embryos in *tg(mpx:GFP/mpeg:mCherry)* background show neutrophils in green and macrophages in red. Embryos were imaged every minute for 7 h; see Movies 1 and 2. The outline of the amputated fin-fold at the start of imaging is indicated with a solid orange line. The border of the wound area is indicated with a dashed orange line. Scale bars: 100 μm. hpa, hours post amputation. (B) Cells were detected using TrackMate. Cells within 200 µm of the wound were counted in every frame from 30 min after amputation to 410 min after amputation. The shaded areas represent the results of bootstrapping. Results of six mutants and 11 siblings were analyzed and compared by ordinary least-squares regression (statmodels). For neutrophils, *P*=0.78 and 95% c.i. coefficient=−2.10 to −1.54; for macrophages, *P*<0.0001 and 95% c.i. coefficient=−0.24 to 0.02. (C) The meandering index and mean speed were determined for the distant cells (further than 200 µm from the wound edge) of six siblings and four mutant embryos. The meandering index was defined as the net distance traveled by the total distance traveled of a track. All results of the measurements in C were compared using unequal variance two-tailed unpaired *t*-test (SciPy) and two-sided 95% c.i. (statmodels). The meandering index was significantly reduced for neutrophils (*P*<0.0001, 95% c.i.=0.48 to 0.52 versus 0.36 to 0.42; and for macrophages, *P*=0.0014, 95% c.i.=0.39 to 0.44 versus 0.45 to 0.49). The mean speed was not significantly different for neutrophils (*P*=0.5, 95% c.i.=7.3 to 8.1 versus 7.4 to 8.5) and macrophages (*P*=0.48, 95% c.i.=4.82 to 5.35 versus 4.89 to 5.61). The number of tracks was not significantly different for the entire tail of six siblings and four mutant embryos (neutrophils, *P*=0.092; macrophages, *P*=0.37). Wound persistence was determined for all cells that entered the wound area of 11 siblings and six mutants. For neutrophils, *P*=0.78, 95% c.i.=0.35 to 0.41 versus 0.35 to 0.42; for macrophages, *P*=0.014, 95% c.i.=0.33 to 0.38 versus 0.28 to 0.34. NS, not significant; **P<0.01; ****P<0.0001. Box plots show quartiles of data, the whiskers show the distribution of the data points within 1.5 times interquartile range, and the median is marked with a line. Outliers were determined as datapoints outside 1.5 interquartile range. The numbers of cells and number of embryos are mentioned as *n*=cells/embryos.

Neutrophils and macrophages play an essential role during injury response. They are among the first cells to be recruited to an injury site, where they remove debris and release molecules that promote inflammation and vasodilation. We investigated whether the behavior of neutrophils and macrophages in *ptpn6* mutant embryos differed from that of siblings by live imaging 4 dpf embryo tails following amputation of the tip of the tail fin fold (Fig. 6A; Movies 1 and 2). The number of neutrophils that migrated into the wound area (closer than 200 µm to the cut site) in mutant embryos was not significantly different from that of their siblings (Fig. 6B). However, the meandering index (net distance traveled/total distance traveled) of neutrophils outside the wound area was lower for mutant embryos than that of their siblings (*P*<0.0001). Speed and wound persistence of neutrophils were not different in mutants lacking functional Shp1 (Fig. 6C).

The total number of macrophages in embryos lacking Shp1 was increased at 3 dpf and 5 dpf (Fig. 4E). In contrast, the number of macrophages in the wound area was significantly decreased in mutant embryos compared to their siblings (*P*<0.0001) (Fig. 6A,B; Movies 1 and 2). This is supported by the finding that wound persistence of macrophages was decreased in mutants compared to siblings (*P*=0.007) (Fig. 6C). The meandering index of macrophages distant from the wound was significantly reduced in mutant embryos (*P*=0.001), whereas the speed was not affected (Fig. 6C). In contrast to the total number of neutrophils and macrophages (Fig. 4E,F), the number of macrophages and neutrophils that were detected in the tail was not significantly different between mutants and siblings, although the number of neutrophils in mutant embryo tails trended towards a reduction (Fig. 6C). Despite the lower number of macrophages in the wound area, there was no difference in regeneration between *ptpn6* mutant embryos and their siblings (Fig. S6). Taken together, embryos lacking functional Shp1 exhibited differences in the behavior of neutrophils and macrophages, which, however, did not affect caudal fin fold regeneration.

## DISCUSSION

SHP1 null mutations have long been studied in the context of autoimmunity and inflammation in the *motheaten* mouse. Because mice develop *in utero* and *motheaten* mice are already severely affected at birth (Green and Shultz, 1975), it has been difficult to study the early development of the hematopoietic system and the inflammatory phenotype. In this study, we developed a zebrafish model to study hematopoiesis during embryonic development in the absence of Shp1. In the mouse embryo, HSCs emerge from the dorsal aorta and colonize the liver before populating the adult organs of hematopoiesis (Sánchez et al., 1996; de Bruijn et al., 2000; Kumaravelu et al., 2002; Boisset et al., 2010). An equivalent process takes place in zebrafish embryos, where HSPCs emerge from the ventral wall of the dorsal aorta, temporarily colonize the CHT, which is homologous to the mouse fetal liver, before migrating to the head kidney, the adult site of hematopoiesis in zebrafish (Kissa and Herbomel, 2010; Murayama et al., 2006). Furthermore, the zebrafish adaptive immune system is not functionally mature until 4-6 weeks post fertilization, allowing analysis of the function of Shp1 in innate immunity during embryonic development.

Shp1 knockout in zebrafish was lethal at the late larval stages, but did not induce obvious morphological defects in embryos. Morpholino-mediated knockdown of Shp1 induced pleiotropic defects from 3 dpf onwards (Kanwal et al., 2013). The pleiotropic defects in response to morpholino-mediated knockdown might be due to off-target effects, causing non-specific inflammation. Alternatively, the difference between the morpholino-mediated knockdown and the knockouts we generated might be caused by intrinsic differences between morpholino-mediated knockdown and genetic knockouts. Morpholinos block translation or splicing from the moment the morpholino is administered, i.e. the one-cell stage, whereas in the genetic knockdown, maternally contributed Shp1 might persist, which might result in a later onset of the phenotype. It is interesting to note that the Shp1 morpholino elicited skin lesions similar to the lesions observed in the genetic mutant (Fig. 2A-D). The inflammatory response was also common between the morpholino-mediated knockdown and the genetic knockout, suggesting that the response to loss of functional Shp1 was similar, but that the timing was different.

Mutant zebrafish larvae present with hyperinflammation, characterized by strong upregulation of several inflammatory genes. The phenotype includes skin lesions and infiltration of gills and mandibular area with neutrophils. The hyperinflammation, the skin lesions and the gill infiltration by neutrophils are very similar to the *motheaten* appearance and lethal pneumonitis found in *motheaten* mice (Green and Shultz, 1975; Jiao et al., 1997). It also supports the sporadic cases of humans that present with mutations in *ptpn6*, which have been associated with neutrophilic dermatoses and emphysema (Nesterovitch et al., 2011; Bossé et al., 2019). The neutrophil-specific Shp1 knockout mouse line suggests that the skin lesions and inflammation are caused by mutant neutrophils. However, the lethal pneumonitis is not recapitulated by neutrophil-specific mouse knockout lines, which indicates that other cell types are involved in causing this phenotype. It is interesting to note that the sites of hyperinflammation are the sites of contact with the outside world, suggesting that external factors might have an important role in the process. The strong occurrence of gill and mandibular infiltration in Shp1 mutant zebrafish makes it an ideal model to investigate the development of lethal pneumonitis/gill infiltration due to Shp1 knockout.

Shp1 deficiency led to multiple hematopoietic defects during embryonic development, affecting HSPCs and myeloid cell lineages. The number of emerging HSPCs was reduced in mutants compared to siblings. However, this reduction was compensated by enhanced proliferation of HSPCs later during development. These results are in contrast with a previous report that showed no effect on HSPCs in *motheaten* mice (Shultz et al., 1983). However, our results support the finding that knockout of Shp1 in HSPCs causes enhanced proliferation in mouse embryos (Jiang et al., 2018). A possible explanation for this apparent discrepancy is that the earlier report missed an effect on HSPCs owing to the use of a spleen assay to determine the number of HSPCs. Enhanced proliferation of HSPCs might have compensated for the lower number of early HSPCs. The zebrafish model facilitated continuous analysis of hematopoiesis from the moment HSPCs emerged from the dorsal aorta, thus revealing reduced numbers of HSPCs at the start, which showed enhanced proliferation later during development (Fig. 5). What drives hyperproliferation of HSPCs remains to be determined definitively. It has been reported in the mouse that loss of SHP1 might lead to enhanced STAT5 phosphorylation, which in turn leads to enhanced proliferation of HSCs (Xiao et al., 2010). It will be interesting to investigate whether Stat5 phosphorylation is enhanced in *ptpn6* mutant zebrafish as well.

The number of macrophages was increased in *ptpn6* mutant zebrafish embryos (Fig. 4), which might simply be due to enhanced numbers of HSPCs in *ptpn6* mutant embryos. The enhanced number of macrophages in *ptpn6* mutant embryos is consistent with earlier findings in *motheaten* and *motheaten viable* mice. Macrophages from the spleen of *motheaten* mice showed increased proliferation and faster maturation than WT macrophages *in vitro* (McCoy et al., 1982; 1983). Tissue sections from *motheaten viable* mice also showed a significant increase in macrophage numbers in spleen, bone marrow and peripheral tissues (Nakayama et al., 1997). Finally, embryonic stem cells expressing dominant-negative Shp1 differentiated into a strongly increased number of myeloid cell colonies in a hematopoietic colony-forming assay (Paling and Welham, 2005). However, the observation that the increase in macrophage number occurred at the expense of the number of neutrophils (Fig. 4) has not been noticed in mice. It would be interesting to investigate the early numbers of neutrophils in mice lacking functional Shp1 to verify whether the phenomenon we noticed in zebrafish is similar in mice.

Directional migration of neutrophils and macrophages after tail fin fold wounding was reduced (Fig. 6; Movies 1 and 2). Furthermore, the number of macrophages that were attracted to the wound site was reduced. This shows that Shp1 has a role in the attraction-migration process that occurs directly after wounding. It is likely that Shp1 has a role in sensing the attraction signal, rather than in sending the signal, given the predominant expression of Shp1 in hematopoietic cells and given the role of Shp1 in intracellular signaling (Plutzky et al., 1992; Abram and Lowell, 2017). It is interesting to note that, even though the meandering index of neutrophils was reduced, this did not lead to a reduction in the number of neutrophils that reached the wound site. Apparently, there was still a strong attraction of neutrophils to the wound site. However, the cells struggled to migrate efficiently towards their target. This is consistent with a previous report that neutrophil adhesion is increased in *motheaten* mice lacking functional SHP1 (Stadtmann et al., 2015). The lower number of macrophages that were present at the wound site indicated that fewer macrophages were attracted to the wound site, which was surprising because the number of macrophages in embryos lacking functional Shp1 was enhanced (Figs 4 and 6). Macrophages were primarily located in the anterior of the embryos, instead of the tail region, which may be due to enhanced adhesion of macrophages in the anterior region of the embryo. The molecular mechanism underlying reduced directional migration of neutrophils and macrophages might involve phosphatidylinositol phosphate levels, which might affect adhesion. SHP1 has a role in PIPKIγ regulation in neutrophils and in the modulation of PI3K in macrophages (Stadtmann et al., 2015; Roach et al., 1998). However, further research is needed to definitively establish the effect of Shp1 on neutrophil and macrophage migration.

Regeneration was unaffected in *ptpn6* mutant zebrafish embryos (Fig. S6), despite the lower number of macrophages that reached the wound site. This is surprising, given a previous report that showed that ablation of macrophages severely impaired regeneration (Li et al., 2012). It is possible that the low number of macrophages that reached the wound site was still sufficient for normal regeneration. Another possibility is that Shp1 knockout affected signaling in macrophages in such a way that fewer macrophages were required for the regeneration response. Taken together, although Shp1 is dispensable for regeneration of the caudal fin fold, it is required for the normal behavior of neutrophils and macrophages. More research is needed to determine the exact role of Shp1 in neutrophil and macrophage function. The *ptpn6* mutant we generated will be instrumental for further research into the function of Shp1 in neutrophils and macrophages.

## MATERIALS AND METHODS

### Zebrafish husbandry

Tübingen long fin (TL) fish were housed and handled according to local guidelines and policies in compliance with national and European law. All procedures involving experimental animals were approved by the local animal experiments committee, Koninklijke Nederlandse Akademie van Wetenschappen-Dierexperimentencommissie (AVD8010020173786). Larvae raised in survival experiments were fed shrimp larval diet with a diameter of 5-50 µm (Caviar, Bernaqua) to control for the possible inability of small larvae to eat the normal feed.

### Generation of the *ptpn6* mutant

Guide RNA (gRNA) was designed using the CHOPCHOP web tool for genome editing (https://chopchop.cbu.uib.no/) and produced according to the protocol of Gagnon et al. (2014) using the published constant reverse oligonucleotide and the following gene-specific forward primer: 5′-TAATACGACTCACTATAGGAACCCTACAGGATAAAGAGTTTTAGAGCTAGAAATAGCAAG-3′. Ribonucleoprotein complexes of N-terminal GFP-labeled Cas9 protein and the gRNA were generated by mixing 10 µg Cas9 protein and 75 ng gRNA in 4 μl buffer (300 mM KCl, final concentration). The complex was injected into WT TL embryos at the one-cell stage. GFP-positive embryos were selected at 6 h post injection and raised to adulthood. F0 was screened for germline mutations by PCR genotyping of the offspring for heterozygous mutations. A 7 bp mutation was identified and the founder was outcrossed twice to TL to obtain a stable line. PCR products of the offspring were sub-cloned in pBluescript sk^−^ (Stratagene) for Sanger sequencing of the mutation. mRNA was isolated from individual embryos of a heterozygous incross at 5 dpf. Reverse transcription and PCR using a forward primer targeting the translation start site at the junction of exons 2 and 3 (5′-ATGGTTCGGTGGTTTCACAGAG-3′) and a reverse primer targeting the junction of exons 6 and 7 (5′-CGTCGAGTAATAGGGCTGTT-3′) allowed sequencing of the 7 bp deletion in *ptpn6* transcripts.

### Genotyping

*Ptpn6* mutations were analyzed by PCR of lysed embryos or fin clips using the following primers targeted to the mutation site: fw, 5′-GGATTCAAAACACAGGGGATTA-3′, and rev, 5′-TTTAACTTGGCAAACACACCTG-3′. PCR products were run on a 4% agarose gel for analysis.

### Polyclonal zebrafish Shp1 antibody

GST-Shp1 fusion protein was produced using a pGex-derived prokaryotic expression vector (ATCC) in *Escherichia coli* B12, followed by glutathione-agarose affinity purification (Sigma-Aldrich). The purified protein was shipped for antibody production (custom polyclonal antibodies, Eurogentec). Rabbit polyclonal anti-Shp1 was purified in house by affinity purification. Briefly, serum was precleared using immobilized GST and a GST fusion protein of highly homologous zebrafish Shp2, to remove antibodies binding to GST and antibodies cross reacting with Shp2. Subsequently, Shp1-specific antibodies were affinity purified using the immobilized GST-Shp1 fusion protein. Before use, zfShp1-specific antibody (1:500 in 5% milk) was incubated with purified zfShp2 protein for 1 h to prevent residual cross reaction with Shp2. The purified antibody was validated by immunoblotting using transfected human embryonic kidney 293 (HEK293) cells expressing zebrafish Shp1.

### Immunoblotting

Prior to snap freezing, fin clips of 5 dpf embryos were collected for genotyping. Three embryos/genotype were pooled and lysed in cold lysis buffer [25 mM HEPES pH 7.4, 150 mM NaCl, 0.25% deoxycholate, 1% Triton X-100, 10 mM MgCl_2_, 1 mM EDTA, 10% glycerol, 1:10 cOmplete mini protease inhibitor cocktail (Roche)] by incubation on ice, followed by sonication (Bioruptor, 15 min, 30 s on/off). Lysates of HEK293T cells expressing zebrafish Shp1 were used as a control. Samples were resolved on a 10% acrylamide gel and transferred to a PVDF membrane. Immunoblotting was performed using Akt-specific (1:500, 9272S, Cell Signaling Technology) and Shp1-specific (1:500) antibodies.

### Confocal, fluorescence and bright-field microscopy

Confocal microscopy was performed on a Leica SP5 microscope. Embryos were anesthetized in tricaine and mounted in 0.8% low-melting agarose in E3 medium (5 mM NaCl, 0.17 mM KCl, 0.33 mM CaCl_2_, 0.33 mM MgCl_2_) in a glass cover dish. For live imaging, mounted embryos were covered in E3 medium containing tricaine. Stereo-fluorescence imaging and bright-field imaging of stained embryos and *in situ* hybridizations were performed on a Leica M165FC microscope connected to a DFC420C camera. Images were processed using ImageJ or Imaris.

### qRT-PCR

Larvae were monitored during raising and were sacrificed at a defined end point (curved, skinny body, not able to swim upright and/or severe skin alterations). The genotype was established by fin clipping, PCR and sequencing. If two or more mutant larvae of the same age were available, healthy siblings in the same tank were picked, genotyped by fin clipping and sacrificed to serve as control samples. Total RNA was extracted from genotyped larvae in TRIzol (Invitrogen). Snap frozen samples were crushed with an Eppendorf tube pestle and further homogenized using a syringe. cDNA was synthesized using SuperScript III First-Strand Synthesis Kit (Invitrogen). qRT-PCR was performed using SYBR green and a CFX-96 Connect Real-Time system (Bio-Rad). Data were analyzed by applying the 2^−ΔΔCt^ method (Livak and Schmittgen, 2001). Primers are listed in Table S1.

### Sudan Black, o-Dianasidine and Acridine Orange staining

3 and 5 dpf embryos were fixed and stained using Sudan Black (Sigma-Aldrich) (Le Guyader et al., 2008) and counted as published previously (Choorapoikayil et al., 2014). o-Dianisidine (Sigma-Aldrich) staining was performed as described before (Iuchi and Yamamoto, 1983), but embryos were fixed afterwards 4% paraformaldehyde) and cleared in 2:1 benzylbenzoate/benzylalcohol. Acridine Orange staining was carried out as described by Choorapoikayil et al. (2014).

### *In situ* hybridization

Embryos were fixed in 4% paraformaldehyde in PBS. *In situ* hybridizations were performed as previously described (Thisse and Thisse, 2008). Published digoxigenin-UTP-labeled riboprobes were used (Choorapoikayil et al., 2014).

### Whole-mount immunohistochemistry

Embryos were fixed overnight in 2% paraformaldehyde. Samples were washed twice in PBS containing 0.1% Tween-20 (PBS-T) followed by 15 min permeabilization in 1 µg/ml proteinase K (Roche) and three washes in PBS-T. Samples were blocked for at least 2 h with 10% goat serum and 0.3% Triton X-100 in PBS, and incubated overnight with chicken anti-GFP antibody (1:500, Aves Labs, GFP-1010) or rabbit anti-pHis3 (1:250, Merck Millipore, 06-570). Embryos were washed ten times for 10 min in PBS containing 0.3% Triton X-100 (PBS-X), followed by 1 h blocking and overnight incubation in either anti-chicken Alexa Fluor 488 (1:500, Invitrogen, A32731) or Cy5 anti-rabbit (1:500, Jackson ImmunoResearch, 711-175-152). The next day, samples were repeatedly washed in PBS-X for 3-4 h and imaged.

### Plasmid construction, RNA synthesis and micro-injections

The coding sequence of zebrafish Shp1 was obtained by PCR (fw, 5′-ACCCTGTTTACGTGTCGAGA-3′; rev, 5′-AGCCTTGGCTCAGTTTTCTT-3′) from a mix of cDNA of 3 and 5 dpf TL embryos and cloned into pCS^2+^eGFP-p2a (RZPD) using In-Fusion cloning (Takara Bio). The R462M point mutation in the PTP domain was introduced by Q5 site-directed mutagenesis (New England Biolabs). pCS^2+^ constructs were linearized using NotI and transcribed *in vitro* (mMessage machine SP6, Ambion). mRNA was injected in one-cell stage embryos at a concentration of 5 ng/µl.

### Tail wounding assays

4 dpf *Tg(mpx:GFP/mpeg:mCherry)* embryos were anesthetized with tricaine and tails were transected distal to the notochord using a scalpel blade. Embryos were immediately mounted for confocal imaging and imaged from ∼30 min post wounding for 7 h every minute. Particles were detected and tracks were analyzed using the Trackmate plugin from Fiji (Tinevez et al., 2017). The linear motion algorithm was used with a maximum allowed gap of four timeframes and a maximum allowed radius of 50 µm. The wound area was defined as 200 μm and closer from the edge of the tail in the first timeframe. The mean speed of tracks outside the wound area was determined by dividing the total distance by the total time of the track. The meandering index of tracks outside the wound area was determined by dividing the net distance (distance between the first and last point of track) by the total distance of the track. Wound persistence was determined for each track that entered the wound area by dividing the number of timeframes spent inside the wound area by the number of timeframes left till the end of the movie.

### Statistical analysis

Analytical statistics were performed in GraphPad Prism 7 (v7.04). For the tail wounding, descriptive statistics were determined using Python (Matplotlib v3.3.2, NumPy v1.18.5, SciPy v1.50, statmodels v0.11.1). For all experiments, samples were genotyped after analysis and split in different experimental groups based on their genotype. Hence, researchers were blinded to the genotype during analysis. All successful images and movies were used for analysis; none were excluded. Sample size was specified to be the maximum number of samples available, as the effect size was unknown prior to the experiment. Statistical tests used in this paper include ANOVA, Tukey's multiple comparisons test, Sidak's multiple comparisons test, Benjamini–Hochberg procedure, log-rank test, ordinary least-squared regression, unequal variance *t*-test and two-sided 95% confidence intervals. Data used for the statistical tests meet the assumptions of the specific test. Specifics of the statistical analyses are indicated in the figure legends.

### Regeneration assay

Zebrafish embryos were amputated at the tail as previously described (Hale et al., 2017). Amputations were performed at 2 dpf and regeneration was analyzed at 5 dpf. Regenerated tails were imaged and whole embryos were lysed for genotyping. Regeneration was quantified by measuring the length from the tip of the notochord to the end of the fin fold in ImageJ.

## Supplementary Material

10.1242/dmm.049715_sup1 Supplementary information

## Data Availability

All relevant data can be found within the article and its supplementary information.
